# Genomic selection of agronomic traits in hybrid rice using an NCII population

**DOI:** 10.1186/s12284-018-0223-4

**Published:** 2018-05-10

**Authors:** Yang Xu, Xin Wang, Xiaowen Ding, Xingfei Zheng, Zefeng Yang, Chenwu Xu, Zhongli Hu

**Affiliations:** 1 Jiangsu Provincial Key Laboratory of Crop Genetics and Physiology, Co-Innovation Center for Modern Production Technology of Grain Crops, Key Laboratory of Plant Functional Genomics of Ministry of Education, Yangzhou University, Yangzhou, 225009 China; 2 State Key Laboratory of Hybrid Rice, College of Life Science, Wuhan University, Wuhan, 430072 China

**Keywords:** Genomic selection, Predictability, Hybrid, Rice, GBLUP

## Abstract

**Background:**

Hybrid breeding is an effective tool to improve yield in rice, while parental selection remains the key and difficult issue. Genomic selection (GS) provides opportunities to predict the performance of hybrids before phenotypes are measured. However, the application of GS is influenced by several genetic and statistical factors. Here, we used a rice North Carolina II (NC II) population constructed by crossing 115 rice varieties with five male sterile lines as a model to evaluate effects of statistical methods, heritability, marker density and training population size on prediction for hybrid performance.

**Results:**

From the comparison of six GS methods, we found that predictabilities for different methods are significantly different, with genomic best linear unbiased prediction (GBLUP) and least absolute shrinkage and selection operation (LASSO) being the best, support vector machine (SVM) and partial least square (PLS) being the worst. The marker density has lower influence on predicting rice hybrid performance compared with the size of training population. Additionally, we used the 575 (115 × 5) hybrid rice as a training population to predict eight agronomic traits of all hybrids derived from 120 (115 + 5) rice varieties each mating with 3023 rice accessions from the 3000 rice genomes project (3 K RGP). Of the 362,760 potential hybrids, selection of the top 100 predicted hybrids would lead to 35.5%, 23.25%, 30.21%, 42.87%, 61.80%, 75.83%, 19.24% and 36.12% increase in grain yield per plant, thousand-grain weight, panicle number per plant, plant height, secondary branch number, grain number per panicle, panicle length and primary branch number, respectively.

**Conclusions:**

This study evaluated the factors affecting predictabilities for hybrid prediction and demonstrated the implementation of GS to predict hybrid performance of rice. Our results suggest that GS could enable the rapid selection of superior hybrids, thus increasing the efficiency of rice hybrid breeding.

**Electronic supplementary material:**

The online version of this article (10.1186/s12284-018-0223-4) contains supplementary material, which is available to authorized users.

.

## Background

The primary mission of rice breeding is to develop high-yield varieties to improve production to meet the global food demand (Xu et al. 2014). Hybrid breeding facilitates to increase rice yield by taking advantage of heterosis, which lead to an approximately 40% increase in rice yield per area during the past 30 years (Beukert et al. 2017). The biggest challenge in hybrid breeding resides in how to efficiently select desired hybrids out of all potential hybrids. In the early days, selection of parental lines largely depends on the experience of breeders, leading to a great deal of uncertainty as well as wasting enormous labor and time in experimental evaluation. Genomic selection (GS) has been proposed as a promising tool to overcome the challenge (Meuwissen et al. 2001). GS can be considered as a novel alternative to traditional marker assisted selection (MAS) for quantitative traits (Hickey et al. 2017). The aim of GS is to combine whole-genome molecular markers and phenotypes in a training population to predict genetic values of future individuals in a test population for selection and no significant test is required, thus avoiding biases in marker effect estimates as well as accelerating the breeding cycle (Desta and Ortiz 2014). Contrary to MAS, GS is suitable for quantitative traits controlled by a large number of small-effect genes. Motivated by the great success in enhancing the rate of genetic gain of livestock breeding, GS has been introduced to plant breeding in many areas, for instance, inbred performance prediction and hybrid prediction (Riedelsheimer et al. 2012a; Crossa et al. 2014; Xu et al. 2014; Wang et al. 2017; Xu 2017).

In hybrid breeding, GS predict the performance of all potential crosses of a given parent set with parents genotyped and a small proportion of crosses evaluated in the field, significantly reducing the cost of hybridization and experimental evaluation of all potential crosses. Although GS has been applied to predict hybrid performance, few GS studies in rice hybrid breeding have been implemented. Xu et al. (2014) provided a proof of concept for hybrid prediction using an immortalized F_2_ population in rice and found that selection of top 100 hybrids would lead to a 16% increase in yield, which indicated the potential of using GS to improve yield in rice. However, the narrow genetic background of the parental materials that derived from the same parents may limit the practical application to rice hybrid breeding. In order to overcome this limitation, we constructed a training population of rice for GS according to the NC II design where 115 inbred varieties were crossed with five male sterile lines. The 120 inbred parents were genotyped and 575 hybrids were measured for eight agronomic traits.

The accurate prediction is essential for successful application of GS. The predictability, representing the accuracy of prediction, obtained from cross-validation in training population has been evaluated in maize, wheat and barley (VanRaden 2008; Crossa et al. 2017). These studies indicated that the predictability is affected by various genetic factors including heritability, relatedness, sample size and marker density, genetic architecture. In general, the predictability increases as marker intensity and sample size increases until reaches a plateau. The marker density required is determined by how quickly linkage disequilibrium (LD) decays in the population. If LD decays slowly, a small number of markers are required to scan the genome, and vice versa (Desta and Ortiz 2014). The relatedness between training population and test populations is also a key factor for predictability. Predictability within full-sib or half-sib families is much higher than that in unrelated groups. In a biparental maize population, including half-sibs from both parents rather than increasing the size of the training population randomly results in an increase in the predictability (Jacobson et al. 2014). The predictability is closely related to the heritability. The traits with higher heritability tend to have higher predictability. The predictabilities of low heritability traits, such as yield, were consistently lower than high heritability traits, such as kernel weight and plant height.

In addition to genetic factors, statistical factors have influence on the predictability. Parametric methods including genomic best linear unbiased prediction (GBLUP) (VanRaden 2008), Bayesian methods (González-Recio and Forni 2011), least absolute shrinkage and selection operator (LASSO) (Tibshirani 1996), partial least squares (PLS) (Gelandi and Kowalski 1986) and nonparametric methods including random forest (Svetnik et al. 2003), neural networks (NN) (Ehret et al. 2015), support vector machine (SVM) (Maenhout et al. 2007) and reproducing kernel Hilbert spaces regression (RKHS) (de Los Campos et al., 2010) have been widely used for GS to predict genetic values. Several investigators have compared the predictive performance of these methods using simulation and empirical data (VanRaden 2008; Riedelsheimer et al. 2012b; Howard et al. 2014; Wang et al. 2015). However, little information exists in comparing the prediction accuracy of such methods in hybrid rice population.

It is worth noting that even if an accurate prediction model is available, it is of no use without superior germplasm resources. Fortunately, the 3000 rice genomes project (3 K RGP) publicly released sequence data of 3023 rice accessions collected from 89 countries, which provided rich germplasm materials for hybrid breeding of rice (Li et al. 2014).

In order to determine the utility of using GS to guide hybrid breeding in rice, we evaluated the accuracy of genomic prediction for hybrid performance in a rice NC II population where 115 inbred varieties were crossed with five male sterile lines using six representative methods including GBLUP, LASSO, BayesB, PLS, SVM and RKHS. The genotypes of 120 inbred parents and eight agronomic traits of 575 hybrids were measured. We also assessed the influence of the statistical method, heritability, marker number and training population size on prediction for predicting hybrid performance. Additionally, we predicted all potential crosses that between the 120 parental lines in the training population and 3023 rice varieties in the 3 K RGP using the optimum prediction models, and finally selected the promising superior hybrids for further hybrid breeding.

## Methods

### Material collection

The hybrid rice population was constructed according to NC II design. A total of 575 hybrids were generated by crossing 115 inbred rice lines with five male sterile lines. Phenotypic data of grain yield per plant (GY), thousand-grain weight (TGW), productive panicle number per plant (PN), plant height (PH), primary branch number (PB), secondary branch number (SB), grain number per panicle (GN), and panicle length (PL) were collected from Wuhan University and Huazhong Agricultural University in 2013. At two locations, ten plants from each cross were planted with a randomized block design in two replicates and the average phenotypic value of each cross from two locations was used in the data analysis.

The 120 parental lines were genotyped using next-generation sequencing and SNPs were called against Nipponbare reference genome (IRGSP-1.0, http://rapdb.dna.affrc.go.jp). Quality control of SNPs was performed by eliminating SNPs with missing rate above 20% in the male sterile lines and above 50% in 115 inbred rice lines. In total, 2,561,889 SNPs remained after this filtering. The genotypic data of 3023 germplasm accessions in 3 K RGP were released. Approximately 20 million SNPs were identified by aligning reads from 3 K RGP with IRGSP-1.0. A total of 6,572,189 SNPs and 996,009 SNPs were selected in 3kRG filtered SNP set v.4 and 3kRG core SNP set v.4, respectively (Zhang et al. 2015). In order to predict potential crosses between the 120 lines and 3023 rice varieties, we aligned our rice SNP dataset with 3kRG filtered SNP set and core SNP set, and then obtained two intersections including 2,054,293 SNPs and 116,482 SNPs, respectively. The genotypes of the hybrids were deduced based on the genotypes of the parental lines.

### Models of prediction

We used four parametric methods including GBLUP, LASSO, PLS, BayesB and two nonparametric methods including RKHS and SVM to predict hybrid performance. The general model of these four parametric methods is described as the following:

1 $$ y= X\beta +\sum \limits_{k=1}^m{Z}_k{\gamma}_k+\varepsilon $$

Where *y* is a vector for *n* observations, *X* is an *n* × *q* design matrix, *β* is a *q* × 1 vector of fixed effects, *m* is the number of markers, *Z*_*k*_ is a column vector for genotype indicator variable, *γ*_*k*_ is additive genetic effect of marker *k*, and *ε* is an *n* × 1 vector of residual errors with an assumed *N*(0, *Iσ*^2^) distribution. The genotypic indicators of marker *k* for individual *j* (where *j* = 1, 2,…, *n*) is defined as − 1, 0, 1 for homozygote of the minor allele, heterozygote and the homozygote of the major allele, respectively. The GBLUP method assumes ${\gamma}_k\sim N\left(0,\frac{1}{m}{\phi}^2\right)$, where *ϕ*^2^ is polygenic variance shared by all makers. The expectation of *y* is E(*y*) = *Xβ* and the variance-covariance matrix is

2 $$ \operatorname{var}(y)=V=\frac{1}{m}\sum \limits_{k=1}^m{Z}_k{Z}_k^T{\phi}^2+I{\sigma}^2=K{\phi}^2+I{\sigma}^2=\left( K\lambda +I\right){\sigma}^2 $$

where *λ* = *ϕ*^2^/*σ*^2^ is the variance ratio and *K* is a marker-generated kinship matrix defined as

3 $$ K=\frac{1}{m}\sum \limits_{k=1}^m{Z}_k{Z}_k^T $$

To estimate the variance components, we used the restricted maximum likelihood method (REML) to maximize the following likelihood function,

4 $$ L\left(\lambda \right)=-\frac{1}{2}\ln \mid V\mid -\frac{1}{2}{\left(y- X\beta \right)}^T{V}^{-1}\left(y- X\beta \right)-\frac{1}{2}\ln \mid {X}^T{V}^{-1}X\mid $$

where *β* is substituted by*β* = (*X*^*T*^*V*^−1^*X*)^−1^*X*^*T*^*V*^−1^*y*and *σ*^2^ is substituted by${\sigma}^2=\frac{1}{n-q}{\left(y- X\beta \right)}^T{V}^{-1}\left(y- X\beta \right)$. The solution of λ was obtained by maximizing the above likelihood function using the newton iteration algorithm. The GBLUP method exploits the genomic relationships between training population and testing population to predict the genomic values for unknown individuals without estimating marker effects. Here, GBLUP was implemented in our own R program.

BayesB is a sampling algorithm based Bayesian approach, which assumes that the prior distribution of variances across markers is a two-component mixture with one component following an inverted chi-square distribution and the other being a point mass at 0 (Meuwissen et al. 2001). It can be summarized as${\gamma}_k\sim N\left(0,\kern0.5em {\sigma}_{\gamma_k}^2\right)$, where ${\sigma}_{\gamma_k}^2=\kern0.5em 0$ with *prob*  =  *π* and ${\sigma}_{\gamma_k}^2\sim {\chi}^{-2}\left(v,S\right)$ with *prob*  = 1 ‐  *π*. The *π*, *v* and *S* are three parameters defined in the original BayesB publication, where *v* and *S* were set to 4.234 and 0.0429, respectively (Meuwissen et al. 2001). In this study, we used “BGLR” package to implement BayesB model and adopted the default values for *v* and *S*. The parameter *π* was unknown and was assigned a weakly informative Beta prior *π* ~ *Beta*(*p*_0_, *π*_0_), with *π*_0_  =  0.5 and *P*_0_ = 10 as default values (Perez and de los Campos 2014).

LASSO is a constrained form of ordinary least squares with a bound on the sum of the absolute values of the coefficients (Tibshirani 1996). The marker effect is defined as

5 $$ {\widehat{\gamma}}_k=\arg \underset{\beta \in \Omega}{\min}\left[{\left(y- X\beta -\sum \limits_{k=1}^m{Z}_k{\gamma}_k\right)}^T\left(y- X\beta -\sum \limits_{k=1}^m{Z}_k{\gamma}_k\right)+\lambda \sum \limits_{k=1}^m|{\gamma}_k|\right] $$

Where *λ* is a shrinkage parameter. LASSO was first implemented in GS by Usai et al. (2009). In this study, LASSO was implemented in an R package called glmnet (Friedman et al. 2010).

The PLS method incorporates principal component analysis (PCA) into multiple regression analysis, and it transforms independent variables into a few linearly uncorrelated components as predictors to predict the phenotype. The number of components was determined by cross-validation to have a minimum prediction error. The PLS method was implemented using an R program called pls (Mevik and Wehrens 2007).

The general form of these two nonparametric methods is defined as:

6 $$ y=\mu +{K}_h\alpha +\varepsilon $$

Where *μ* is the population mean; *K*_*h*_ is a kernel function, which can be used to map the input data to a high-dimensional space where the data can be more easily separated; *α* and *ε* are assumed to have independent prior distributions $\alpha \sim N\left(0,{K}_h{\sigma}_{\alpha}^2\right)$ and *ε*~*N*(0, *Iσ*^2^). The SVM method is a kernel based supervised learning method for classification and regression, and Maenhout et al. (2007) first applied it in GS to predict maize hybrid performance. Several kernel functions, such as polynomial, Gaussian radial basis function, and the linear kernel, have been commonly used in SVM. Here, we chose the Gaussian kernel function and implemented it in an R package kernlab (Karatzoglou et al. 2004).

RKHS has been used for spatial smoothing, regression and classification, in which the reproducing kernel (RK) is one of the central elements of model specification. Here, we selected the multi-kernel function and implemented the method in the R package BGLR.

### Predictability and heritability

The predictability for rice hybrid performance was evaluated using a fivefold cross-validation, where the sample was randomly partitioned into five parts with four parts being used to estimate parameters and the remaining part being predicted. Finally, all parts were predicted once and used four times to estimate parameters. The predictability is defined as the correlation coefficient between the observed and predicted phenotypic values. The predictability may be affected by how the sample is partitioned into the fivefold. Therefore, we replicated the cross-validation analysis 20 times to achieve the average prediction results of these replicates. In order to identify the impacts of training population size and marker number on predictability, we used different subsets of training population and markers to evaluate the predictability.

Broad-sense heritability (*H*) can be calculated as described by Knapp et al. (1985): $H={\sigma}_g^2/\left({\sigma}_g^2+{\sigma}_{ge}^2/e+{\sigma}_{\varepsilon}^2/ er\right)$, where ${\sigma}_g^2$ is the genetic variance, ${\sigma}_{ge}^2$ is genotype-by-environment interaction variance, ${\sigma}_{\varepsilon}^2$ is residual variance, *e* is the number of environments and *r* is the number of replications in each experiment. Here, we calculated the broad-sense heritability based on the phenotypic data collected from two locations with two replications using analysis of variance (ANOVA).

## Results

### Comparison of predictabilities

**Fig. 1:**
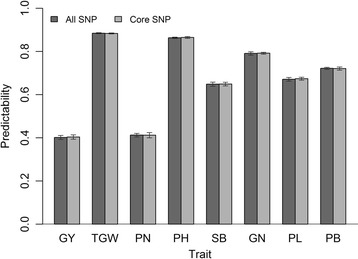
Predictabilities of eight traits from two subsets of SNPs. All SNP defines the intersection of our rice SNP dataset and 3kRG filtered SNP set, including 2,054,293 SNPs; Core SNP defines the intersection of our rice SNP dataset and core SNP set, including 116,482 SNPs. Error bars are constructed using one standard error from the mean

By comparing our rice SNP dataset with 3kRG filtered SNP set and 3kRG core SNP set, we obtained two rice SNP intersections, all SNP set and core SNP set, both of which were used to evaluate the accuracy of prediction. The predictabilities of eight traits obtained from the GBLUP method are illustrated in Fig. 1. Although the number of markers in all SNP set (2,054,293) is much larger than that in core SNP set (116,482), the predictabilities of using these two SNP datasets are nearly the same for all the eight traits. To improve computational efficiency, we used core SNP set in the follow-up analyses.

The predictabilities of the eight traits obtained from 575 hybrids using all the six methods including GBLUP, LASSO, PLS, BayesB, RKHS and SVM are summarized in Table 1. The predictability is highly correlated to the heritability of the trait with TGW and PH having the highest predictabilities across all methods, followed by traits PL and SB, with trait GY and PN being the worst predictable traits. The predictabilities of most traits are larger than 0.5, except GY and PN. For the same trait, the largest differences in predictability among the six methods vary from 0.007 to 0.046. Standard deviations of predictabilities range from 0.0024 and 0.015 across traits and methods, where the high predictable traits tend to have smaller standard deviations than those low predictable traits.

**Table 1:** Average predictabilities and their standard deviations (sd) for eight traits using six prediction methods

Trait	Heritability	GBLUP		PLS		LASSO		BayesB		SVM		RKHS	
		mean	sd	mean	sd	mean	sd	mean	sd	mean	sd	mean	sd
GY	0.3031	0.4057	0.0102	0.3832	0.0125	0.3923	0.0132	0.4095	0.0117	0.4032	0.012	0.4041	0.0122
TGW	0.8501	0.8833	0.0025	0.8735	0.0037	0.8821	0.0024	0.8819	0.0035	0.8791	0.0037	0.8829	0.0029
PN	0.2550	0.4122	0.0122	0.3849	0.0141	0.4156	0.015	0.4189	0.011	0.3731	0.0125	0.3975	0.0114
PH	0.7501	0.8647	0.0041	0.8564	0.0048	0.8642	0.0038	0.8629	0.0046	0.8644	0.0064	0.8601	0.0043
SB	0.6676	0.7158	0.0072	0.7085	0.0121	0.7181	0.0061	0.7122	0.0076	0.6862	0.0112	0.715	0.0081
GN	0.6262	0.6488	0.0085	0.6356	0.0093	0.6445	0.0089	0.6451	0.0095	0.6197	0.0133	0.6478	0.0094
PL	0.7802	0.7919	0.0045	0.7897	0.0049	0.7922	0.0041	0.7887	0.0044	0.7957	0.0043	0.7894	0.0048
PB	0.6944	0.6736	0.0069	0.6613	0.0077	0.6857	0.0062	0.6739	0.0076	0.6761	0.0058	0.6746	0.0066

*Abbreviations*: *GY* Grain yield per plant, *TGW* Thousand-grain weight, *PN* Productive panicle number per plant, *PH* Plant height, *SB* Secondary branch number, *GN* Grain number per panicle, *PL* Panicle length, *PB* Primary branch number

Predictabilities are averaged over 20 cross-validation runs

### Analysis of variance for predictability

**Fig. 2:**
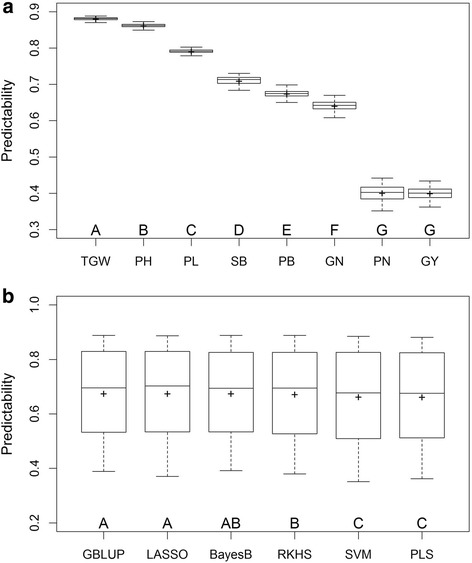
Multiple comparisons illustrated by boxplots. In each panel, different capital letters above the group labels indicate significant differences between groups. In each boxplot, the cross sign represents the mean predictability. Panel a compares the predictabilities for the eight traits over six methods and 20 replications. Panel b compares the predictabilities of the six methods across eight traits and 20 replications

We performed analyses of variances for predictabilities of eight traits and six methods over 20 repetitions. All main effects and interaction effects are significant (Table 2). Then we performed multiple comparisons for the main effects and the results are presented in Fig. 2. Predictabilities of the eight traits are significantly different with TGW being the best, and PN and GY being the worst (Fig. 2a). Among the six methods, GBLUP and LASSO perform the best, followed by BayesB and RKHS, and SVM and PLS perform the worst (Fig. 2b). Although GBLUP and LASSO possess overall good performance, different methods may be suitable for different traits. From Additional file 1: Table S1, we can find that GBLUP is the most efficient method for traits GN, PH and TGW while LASSO is the most efficient for traits PB and SB. SVM is the best method for trait PL, whereas it is the worst for traits GN, SB and PN. BayesB performs the best for GY and PN but the worst for PL. PLS performs poorly for most traits.

**Table 2:** Analyses of variances of predictabilities for eight traits and six prediction methods with 20 replications

Source	DF	Sum of Square	Mean Square	F-test	*p*-value
Trait	7	29.2992	4.1856	57,118.62	<.0001
Method	5	0.0302	0.0060	82.35	<.0001
Method×Trait	35	0.0492	0.0014	19.19	<.0001
Residual	912	0.0668	7.33E-05

### Influence of marker number and training population size on predictability

**Fig. 3:**
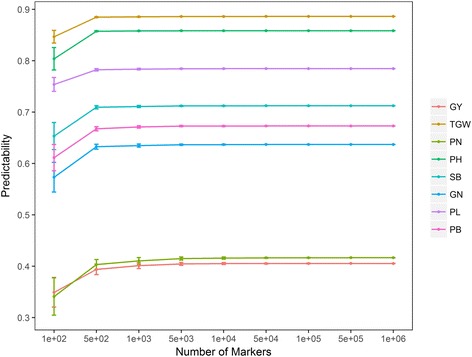
Effect of the marker density on the predictability. Nine subsets are selected with the marker number varying from 100 to 1,000,000. Fivefold cross-validations are repeated 100 times for each subset of SNP markers. Error bars are constructed using one standard error from the mean

In order to determine the effect of marker density on performing GS in a rice population of this kind, we selected nine SNP subsets with the number of markers varying from one hundred to one million. One hundred selections in a random way were made for each subset size. From Fig. 3, it is clear that there is nearly no difference in the predictability for each trait whether one million SNPs are used or 5000 SNPs are used. When the number of markers falls below one thousand, the predictabilities begin to decrease significantly for all traits. The result also reveals that the smaller the number of markers, the larger the variation in predictabilities.

**Fig. 4:**
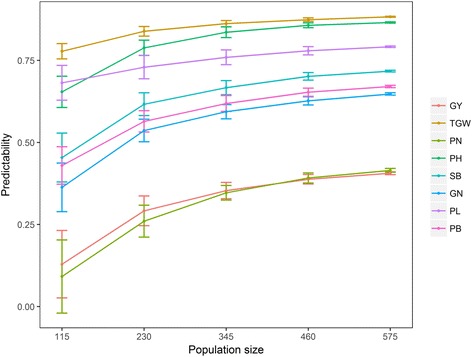
Effect of the population size on the predictability. Five subsets are selected with the number of individuals varying from 115 to 575. Fivefold cross-validations are repeated 100 times for each subset of the training population. Error bars are constructed using one standard error from the mean

The impact of population size was also investigated using the similar strategy. We selected five subsets varying from 115 individuals to the full 575 individuals. As the number of individuals increases, we can observe an increase in the predictability and a decrease in the variation of predictions for each trait (Fig. 4). The population size has larger effects on predicting GY and PN than other traits. As the size of training population decreases from 575 to 115, the predictabilities for PN and GY drop by 77.94% and 68.23%, respectively. Although both the marker density and the size of training population have influences on the predictability, the influence of population size on the predictability is considerably greater than that of the marker density. For example, as the number of makers decreases from one million to one hundred, the predictabilities of eight traits only decline by 9.27% on average, whereas the predictabilities drop by 39.11% on average as the population size decreases from 575 to 115, which indicates that a large training population is necessary to obtain high predictability.

### Predicting untested crosses using GBLUP

**Fig. 5:**
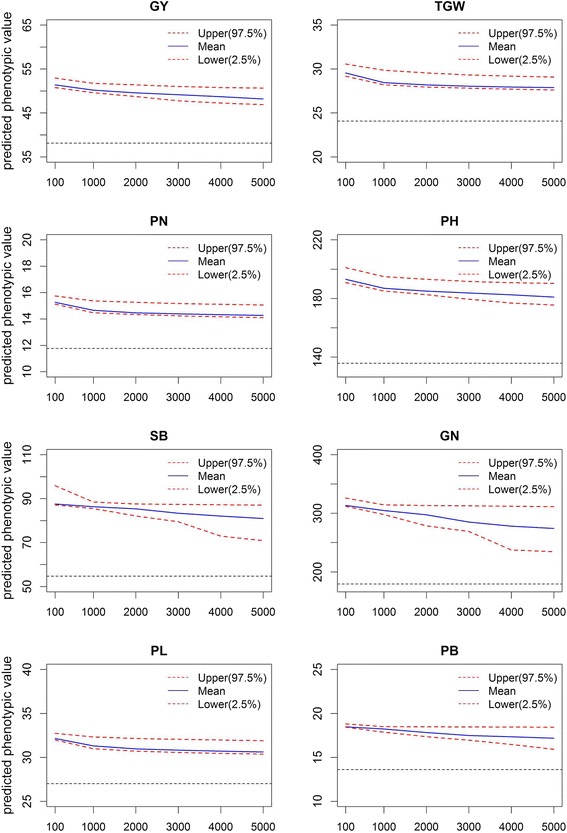
Predicted phenotypic values of selected top crosses plotted against the number of crosses selected. The two red dotted curves define the 95% confidence intervals of the mean predicted phenotypic values. The black horizontal line in each panel denotes the average predicted phenotypic value of all crosses for that trait. The plot is truncated at 5000, and the total number of top crosses is 362,760

The high predictabilities of the eight traits obtained from fivefold cross-validation indicate that genomic selection will be effective for all traits, especially for TGW and PH. According to the parameters estimated from this training sample, we predicted all potential hybrids that between the 120 inbred lines in the training population and 3023 rice varieties in the 3 K RGP using the GBLUP method. Afterwards, we sorted all of the predicted phenotypic values in descending order and averaged the selected top crosses to observe the gains of prediction. Figure 5 shows the average predicted phenotypic values for each trait against different numbers of top crosses. The average predicted phenotypic values of the top crosses are much higher than those of all potential crosses for all traits. For example, the average predicted GY of the top 100 crosses is 51.46, while that of all crosses is 38.13. If the top 100 crosses for GY are selected for hybrid breeding, GY will increase by 13.33 (51.46–38.13), representing a 34.97% (13.33/38.13) gain. For the other seven traits, with genomic selection of the top 100 crosses, we expect to gain 23.25%, 30.21%, 42.87%, 61.80%, 75.83%, 19.24% and 36.12% in TGW, PN, PH, SB, GN, PL and PB, respectively. The combinations of top 100 crosses for the eight traits are given in Additional file 2: Table S2. Breeders can produce these crosses in the field according to these results.

## Discussion

In this study, we evaluated the influences of the statistical method, heritability, marker number and training population size on prediction for hybrid performance in rice. From the comparison of different prediction methods, we found that parametric methods (GBLUP, LASSO, BayesB) performed better than nonparametric methods (RKHS and SVM), but there was no method that fitted all traits universally well. Previous studies have compared the performance of the parametric models with the nonparametric models used in GS. Heslot et al. (2012) compared six parametric methods and four nonparametric methods for genomic prediction in wheat, maize and barley, and observed that the RKHS method performed the best across different species. Howard et al. (2014) assessed 14 parametric and nonparametric methods using simulated genetic architectures, and found that parametric methods performed slightly better than nonparametric methods for additive genetic architectures, but parametric methods had difficulty in capturing non-additive effects such as epistatic effects. Generally, GBLUP is the most robust method and generally gives the higher predictability for highly polygenic traits; the Bayesian methods and LASSO are better for traits with major genes; the PLS method fits data better when individual predictors are highly correlated; SVM and RKHS perform well for traits under non-additive genetic architectures. Some studies have confirmed that small gains in predictability can be obtained if the GS method is selected according to the trait architecture (Riedelsheimer et al. 2012b). If the genetic architecture underlying the trait is unclear, both parametric and nonparametric methods should be tried to cross-confirm the results.

Although the six GS methods differ slightly in their predictabilities, the selected top individuals are different. To verify it, we predicted GY and TGW of the 362,760 hybrids using the LASSO and GBLUP and then compared the prediction results of these two methods. The combinations of top 100 crosses selected based on LASSO for GY and TGW are given in Additional file 3: Table S3-S4. The correlation coefficients between LASSO and GBLUP for predicted GY and TGW are 0.908 and 0.939, respectively. However, among the top 100 crosses selected using LASSO and GBLUP, only 21 and 61 crosses are identical for GY and TGW, respectively (marked in yellow in Table S3-S4). To ensure the reliability of GS, we suggest to choose elite hybrids selected by multiple GS methods.

We also found that the size of training population had a greater impact on predicting hybrid performance than the marker density did, which was in accordance with earlier studies. The increase in predictability quickly reaches a plateau as the number of markers increases. In our study, the predictability plateaued when 5000 markers were used for prediction of all traits. Research in an elite rice breeding population genotyped with 73,147 markers revealed that prediction accuracy reached a plateau at 7142 SNPs with the rrBLUP method (Spindel et al. 2015). Therefore, a low-density marker panel is desired to obtain a favorable cost-benefit ratio for GS. With respect to the size of training population, it has strong effect on the predictability. We observed a monotonic increase in the predictability for each trait with enhancing population size. For GY and PN, the predictabilities obtained from 575 individuals are almost four times as high as those obtained from 115 individuals. Therefore, increasing the size of training population rather than increasing the marker number can be preferable for rice hybrid prediction.

Currently, researches on GS are mainly based on the additive model. In this study, the additive model was used to predict hybrid performance in rice. However, few studies have suggested that incorporating dominance can produce similar or higher predictability than only considering additive effects (Vitezica et al. 2013). Here, we investigated the effects of additive and dominant variances on the prediction of rice hybrid performance. The predictabilities under additive and additive-dominant model for eight traits were evaluated using the HAT method (Xu 2017). The variances were estimated using the REML analysis. The predictabilities and estimated variance components are listed in Additional file 4: Table S5. The result reveals that additive variances explain the majority of the trait variances, and the improvement in predictability by including dominance variances is marginal. This result is consistent with Xu et al. (2014) who did not find benefit from adding non-additive variance. This may be because the kinship matrix of additive effect has already captured much information about the kinship matrix of dominant effect. In consideration of computation efficiency, it may not be necessary to use additive-dominant model to predict hybrid performance in rice.

This study demonstrated the application of GS for predicting the hybrid performance in rice. We used 575 existing hybrids derived from 120 inbred parents as the training population to predict all 362,760 potential hybrids that between the 120 inbred lines and 3023 rice varieties in the 3 K RGP. Only the phenotypes of the 575 hybrids and genotypes of the inbred parents were measured, which enormously reduced the cost of sequencing and experimental evaluation of all potential crosses. Of all the potential hybrids, selection of the top 100 predicted hybrids would lead to a 35% gain in grain yield. Xu et al. (2014) predicted that if the top 100 crosses of 21,945 hybrids were selected for yield, the gain would be 16%. This high gain for the yield with low predictability is mainly due to the high selection intensity of the crosses, represented by the considerably small proportion selected. Theoretically, the selected top crosses can bring a substantial improvement of future hybrids, but these need to be further tested and validated in designed experiments. We are planning to generate the hybrids of the predicted top, middle and last 50 hybrids from all the potential hybrids and evaluate their performance in the field. Then, breeders can select and produce the ideal crosses based on the results of the present study and their experience.

## Conclusions

We used a rice NCII population consisting of 575 hybrids to evaluate the genetic and statistical factors affecting hybrid prediction in rice. The results showed that predictabilities for different methods were significantly different, with the GBLUP and LASSO methods being better than the other methods. The size of training population had greater influence on prediction for rice hybrid performance compared with the marker density. Additionally, selection of the top 100 crosses from all potential hybrids that between the 120 inbred lines in the NCII population and 3023 rice varieties in the 3 K RGP will lead to substantial increase in yield. Our results hold great promise for the implementation of GS in rice hybrid breeding.

## Additional files

Additional file 1: **Table S1.** Multiple comparisons of six prediction methods for eight traits. (DOCX 16 kb)

Additional file 2: **Table S2.** The predicted top 100 crosses for eight agronomic traits. (XLSX 33 kb)

Additional file 3: **Table S3.** The predicted top 100 crosses for grain yield using LASSO. **Table S4.** The predicted top 100 crosses for thousand-grain weight using LASSO (DOCX 19 kb)

Additional file 4: **Table S5**. Estimated variances and predictabilities under additive model and additive-dominance model. (DOCX 14 kb)

## Abbreviations

3 K RGP: 3000 rice genomes project
GBLUP: Genomic best linear unbiased prediction
GN: Grain number per panicle
GS: Genomic selection
GY: Grain yield per plant
LASSO: Least absolute shrinkage and selection operation
LD: Linkage disequilibrium
MAS: Marker assisted selection
NC II: North Carolina II
PB: Primary branch number
PH: Plant height
PL: Panicle length
PLS: Partial least square
PN: Productive panicle number per plant
RKHS: Reproducing kernel Hilbert space
SB: Secondary branch number
SVM: Support vector machine
TGW: Thousand-grain weight
