# Supplementary material for: Genomic selection of agronomic traits in hybrid rice using an NCII population
Source: Rice (N Y). 2018 May 10;11:32. doi: 10.1186/s12284-018-0223-4 (PMC5945574; doi:10.1186/s12284-018-0223-4)
Supplement: Supplementary file 1 — Table S1. Multiple comparisons of six prediction methods for eight traits. (DOCX 16 kb) [file 12284_2018_223_MOESM1_ESM.docx]

Table S1. Multiple comparisons of six prediction methods for eight traits

| Trait | Method | N | Predictability | | Group |
| --- | --- | --- | --- | --- | --- |
|  |  |  | Mean | Std Dev |  |
| GN | GBLUP | 20 | 0.6488 | 0.0085 | A |
|  | RKHS | 20 | 0.6478 | 0.0094 | A |
|  | BayesB | 20 | 0.6451 | 0.0095 | A |
|  | LASSO | 20 | 0.6445 | 0.0089 | AB |
|  | PLS | 20 | 0.6356 | 0.0093 | BC |
|  | SVM | 20 | 0.6197 | 0.0133 | C |
| GY | BayesB | 20 | 0.4095 | 0.0117 | A |
|  | GBLUP | 20 | 0.4057 | 0.0102 | A |
|  | RKHS | 20 | 0.4041 | 0.0122 | A |
|  | SVM | 20 | 0.4032 | 0.0120 | AB |
|  | LASSO | 20 | 0.3923 | 0.0132 | BC |
|  | PLS | 20 | 0.3832 | 0.0125 | C |
| PB | LASSO | 20 | 0.6857 | 0.0062 | A |
|  | SVM | 20 | 0.6761 | 0.0058 | B |
|  | RKHS | 20 | 0.6746 | 0.0066 | B |
|  | BayesB | 20 | 0.6739 | 0.0076 | B |
|  | GBLUP | 20 | 0.6736 | 0.0069 | B |
|  | PLS | 20 | 0.6613 | 0.0077 | C |
| PH | GBLUP | 20 | 0.8647 | 0.0041 | A |
|  | SVM | 20 | 0.8644 | 0.0064 | AB |
|  | LASSO | 20 | 0.8642 | 0.0038 | AB |
|  | BayesB | 20 | 0.8629 | 0.0046 | AB |
|  | RKHS | 20 | 0.8601 | 0.0043 | BC |
|  | PLS | 20 | 0.8564 | 0.0048 | C |
| PL | SVM | 20 | 0.7957 | 0.0043 | A |
|  | LASSO | 20 | 0.7922 | 0.0041 | AB |
|  | GBLUP | 20 | 0.7919 | 0.0045 | AB |
|  | PLS | 20 | 0.7897 | 0.0049 | B |
|  | RKHS | 20 | 0.7894 | 0.0048 | B |
|  | BayesB | 20 | 0.7887 | 0.0044 | B |
| PN | BayesB | 20 | 0.4189 | 0.0110 | A |
|  | LASSO | 20 | 0.4156 | 0.0150 | A |
|  | GBLUP | 20 | 0.4122 | 0.0122 | A |
|  | RKHS | 20 | 0.3975 | 0.0114 | B |
|  | PLS | 20 | 0.3849 | 0.0141 | C |
|  | SVM | 20 | 0.3731 | 0.0125 | C |
| SB | LASSO | 20 | 0.7181 | 0.0061 | A |
|  | GBLUP | 20 | 0.7158 | 0.0072 | AB |
|  | RKHS | 20 | 0.7150 | 0.0081 | AB |
|  | BayesB | 20 | 0.7122 | 0.0076 | AB |
|  | PLS | 20 | 0.7085 | 0.0121 | B |
|  | SVM | 20 | 0.6862 | 0.0112 | C |
| TGW | GBLUP | 20 | 0.8833 | 0.0025 | A |
|  | RKHS | 20 | 0.8829 | 0.0029 | A |
|  | LASSO | 20 | 0.8821 | 0.0024 | A |
|  | BayesB | 20 | 0.8819 | 0.0035 | AB |
|  | SVM | 20 | 0.8791 | 0.0037 | B |
|  | PLS | 20 | 0.8735 | 0.0037 | C |
