# Supplementary material for: Genomic selection of agronomic traits in hybrid rice using an NCII population
Source: Rice (N Y). 2018 May 10;11:32. doi: 10.1186/s12284-018-0223-4 (PMC5945574; doi:10.1186/s12284-018-0223-4)
Supplement: Supplementary file 3 — Table S3. The predicted top 100 crosses for grain yield using LASSO. Table S4. The predicted top 100 crosses for thousand-grain weight using LASSO (DOCX 19 kb) [file 12284_2018_223_MOESM3_ESM.docx]

Table S3. The predicted top 100 crosses for grain yield using LASSO

| Order | Combinations | GY | Order | Combinations | GY |
| --- | --- | --- | --- | --- | --- |
| 1 | IRIS_313-9600/V93 | 54.362 | 51 | IRIS_313-10268/V93 | 52.572 |
| 2 | CX117/V93 | 54.322 | 52 | CX126/V93 | 52.544 |
| 3 | IRIS_313-10307/V93 | 54.218 | 53 | CX265/V93 | 52.544 |
| 4 | CX118/V93 | 54.160 | 54 | CX118/V105 | 52.543 |
| 5 | IRIS_313-10753/V93 | 54.102 | 55 | IRIS_313-7809/V93 | 52.537 |
| 6 | IRIS_313-7635/V93 | 53.842 | 56 | IRIS_313-11233/V93 | 52.536 |
| 7 | B146/V93 | 53.807 | 57 | B193/V93 | 52.529 |
| 8 | IRIS_313-11665/V93 | 53.529 | 58 | IRIS_313-11465/V93 | 52.526 |
| 9 | IRIS_313-11340/V93 | 53.483 | 59 | IRIS_313-11357/V93 | 52.521 |
| 10 | B125/V93 | 53.327 | 60 | IRIS_313-10753/V105 | 52.485 |
| 11 | CX357/V93 | 53.247 | 61 | IRIS_313-10785/V93 | 52.484 |
| 12 | CX18/V93 | 53.197 | 62 | IRIS_313-10560/V93 | 52.482 |
| 13 | IRIS_313-11974/V93 | 53.166 | 63 | IRIS_313-8214/V93 | 52.478 |
| 14 | B151/V93 | 53.158 | 64 | IRIS_313-11868/V93 | 52.422 |
| 15 | B181/V93 | 53.096 | 65 | IRIS_313-11517/V93 | 52.420 |
| 16 | CX122/V93 | 53.077 | 66 | IRIS_313-7635/V43 | 52.416 |
| 17 | IRIS_313-10000/V93 | 53.053 | 67 | CX542/V93 | 52.416 |
| 18 | IRIS_313-9363/V93 | 53.047 | 68 | IRIS_313-10404/V93 | 52.402 |
| 19 | IRIS_313-10910/V93 | 53.021 | 69 | B146/V43 | 52.380 |
| 20 | IRIS_313-11866/V93 | 52.991 | 70 | IRIS_313-9002/V93 | 52.380 |
| 21 | IRIS_313-9600/V43 | 52.936 | 71 | IRIS_313-10164/V93 | 52.380 |
| 22 | IRIS_313-11418/V93 | 52.924 | 72 | B104/V93 | 52.378 |
| 23 | CX250/V93 | 52.922 | 73 | B171/V93 | 52.376 |
| 24 | CX92/V93 | 52.900 | 74 | CX17/V93 | 52.358 |
| 25 | CX117/V43 | 52.896 | 75 | B138/V93 | 52.334 |
| 26 | IRIS_313-11563/V93 | 52.882 | 76 | IRIS_313-10831/V93 | 52.322 |
| 27 | IRIS_313-10307/V43 | 52.792 | 77 | IRIS_313-9375/V93 | 52.319 |
| 28 | CX394/V93 | 52.782 | 78 | IRIS_313-10740/V93 | 52.298 |
| 29 | IRIS_313-8681/V93 | 52.749 | 79 | CX369/V93 | 52.277 |
| 30 | IRIS_313-9600/V105 | 52.745 | 80 | CX273/V93 | 52.260 |
| 31 | B121/V93 | 52.744 | 81 | CX247/V93 | 52.252 |
| 32 | CX118/V43 | 52.734 | 82 | IRIS_313-8170/V93 | 52.251 |
| 33 | CX421/V93 | 52.727 | 83 | IRIS_313-11733/V93 | 52.249 |
| 34 | B114/V93 | 52.705 | 84 | IRIS_313-12045/V93 | 52.237 |
| 35 | CX117/V105 | 52.705 | 85 | IRIS_313-7635/V105 | 52.225 |
| 36 | IRIS_313-11339/V93 | 52.704 | 86 | IRIS_313-11990/V93 | 52.218 |
| 37 | IRIS_313-9570/V93 | 52.704 | 87 | IRIS_313-10699/V93 | 52.213 |
| 38 | IRIS_313-11946/V93 | 52.692 | 88 | IRIS_313-10741/V93 | 52.192 |
| 39 | CX362/V93 | 52.691 | 89 | IRIS_313-12234/V93 | 52.190 |
| 40 | IRIS_313-11012/V93 | 52.690 | 90 | B146/V105 | 52.190 |
| 41 | IRIS_313-10753/V43 | 52.676 | 91 | IRIS_313-8433/V93 | 52.184 |
| 42 | B040/V93 | 52.661 | 92 | IRIS_313-11738/V93 | 52.156 |
| 43 | CX328/V93 | 52.655 | 93 | B234/V93 | 52.145 |
| 44 | B067/V93 | 52.639 | 94 | CX49/V93 | 52.143 |
| 45 | IRIS_313-9423/V93 | 52.629 | 95 | B156/V93 | 52.119 |
| 46 | IRIS_313-8084/V93 | 52.613 | 96 | B060/V93 | 52.115 |
| 47 | IRIS_313-10307/V105 | 52.601 | 97 | CX280/V93 | 52.111 |
| 48 | CX343/V93 | 52.600 | 98 | IRIS_313-7820/V93 | 52.106 |
| 49 | IRIS_313-11884/V93 | 52.597 | 99 | IRIS_313-11665/V43 | 52.103 |
| 50 | IRIS_313-11815/V93 | 52.595 | 100 | IRIS_313-11270/V93 | 52.101 |

Table S4. The predicted top 100 crosses for thousand-grain weight using LASSO

| Order | Combinations | TGW | Order | Combinations | TGW |
| --- | --- | --- | --- | --- | --- |
| 1 | CX250/V96 | 30.342 | 51 | CX290/V96 | 29.000 |
| 2 | IRIS_313-7635/V96 | 30.047 | 52 | B146/V104 | 28.988 |
| 3 | CX250/V101 | 29.985 | 53 | B239/V96 | 28.985 |
| 4 | B242/V96 | 29.856 | 54 | CX370/V104 | 28.983 |
| 5 | CX303/V96 | 29.792 | 55 | B248/V96 | 28.982 |
| 6 | B146/V96 | 29.745 | 56 | CX83/V96 | 28.977 |
| 7 | CX370/V96 | 29.740 | 57 | CX101/V96 | 28.968 |
| 8 | IRIS_313-7635/V101 | 29.690 | 58 | IRIS_313-7635/V100 | 28.968 |
| 9 | CX250/V104 | 29.585 | 59 | B146/V30 | 28.960 |
| 10 | IRIS_313-11568/V96 | 29.581 | 60 | IRIS_313-11731/V96 | 28.957 |
| 11 | CX250/V30 | 29.557 | 61 | CX370/V30 | 28.956 |
| 12 | B242/V101 | 29.500 | 62 | B242/V95 | 28.956 |
| 13 | IRIS_313-12207/V96 | 29.459 | 63 | CX250/V106 | 28.949 |
| 14 | CX250/V95 | 29.441 | 64 | CX250/V115 | 28.946 |
| 15 | CX303/V101 | 29.435 | 65 | CX273/V96 | 28.928 |
| 16 | B238/V96 | 29.415 | 66 | B242/V109 | 28.922 |
| 17 | CX250/V109 | 29.408 | 67 | IRIS_313-11296/V96 | 28.914 |
| 18 | IRIS_313-12190/V96 | 29.389 | 68 | B097/V96 | 28.913 |
| 19 | B146/V101 | 29.388 | 69 | CX51/V96 | 28.911 |
| 20 | CX370/V101 | 29.384 | 70 | CX145/V101 | 28.898 |
| 21 | CX250/V94 | 29.353 | 71 | CX431/V101 | 28.897 |
| 22 | IRIS_313-7635/V104 | 29.290 | 72 | CX303/V95 | 28.891 |
| 23 | CX250/V100 | 29.263 | 73 | CX250/V92 | 28.889 |
| 24 | IRIS_313-7635/V30 | 29.262 | 74 | CX274/V96 | 28.884 |
| 25 | CX145/V96 | 29.255 | 75 | IRIS_313-7635/V56 | 28.883 |
| 26 | CX431/V96 | 29.254 | 76 | B242/V94 | 28.867 |
| 27 | IRIS_313-11568/V101 | 29.225 | 77 | CX342/V101 | 28.865 |
| 28 | CX342/V96 | 29.221 | 78 | CX303/V109 | 28.858 |
| 29 | CX250/V56 | 29.178 | 79 | IRIS_313-7635/V79 | 28.850 |
| 30 | IRIS_313-7635/V95 | 29.146 | 80 | IRIS_313-11719/V96 | 28.846 |
| 31 | CX250/V79 | 29.145 | 81 | B146/V95 | 28.844 |
| 32 | CX131/V96 | 29.133 | 82 | CX250/V97 | 28.843 |
| 33 | CX84/V96 | 29.130 | 83 | CX370/V95 | 28.839 |
| 34 | IRIS_313-11979/V96 | 29.127 | 84 | CX250/V102 | 28.839 |
| 35 | CX314/V96 | 29.120 | 85 | CX231/V96 | 28.832 |
| 36 | IRIS_313-7635/V109 | 29.113 | 86 | IRIS_313-9602/V96 | 28.828 |
| 37 | IRIS_313-12207/V101 | 29.102 | 87 | IRIS_313-11941/V96 | 28.827 |
| 38 | B242/V104 | 29.099 | 88 | IRIS_313-11568/V104 | 28.824 |
| 39 | CX387/V96 | 29.093 | 89 | CX250/V107 | 28.824 |
| 40 | IRIS_313-7826/V96 | 29.074 | 90 | CX123/V96 | 28.812 |
| 41 | B242/V30 | 29.072 | 91 | B146/V109 | 28.810 |
| 42 | CX296/V96 | 29.072 | 92 | CX250/V78 | 28.807 |
| 43 | B238/V101 | 29.059 | 93 | CX370/V109 | 28.806 |
| 44 | IRIS_313-7635/V94 | 29.058 | 94 | IRIS_313-11122/V96 | 28.806 |
| 45 | CX250/V108 | 29.048 | 95 | CX303/V94 | 28.803 |
| 46 | CX303/V104 | 29.035 | 96 | CX343/V96 | 28.799 |
| 47 | IRIS_313-12190/V101 | 29.033 | 97 | IRIS_313-11568/V30 | 28.797 |
| 48 | B138/V96 | 29.018 | 98 | B242/V100 | 28.777 |
| 49 | CX303/V30 | 29.007 | 99 | CX8/V96 | 28.777 |
| 50 | IRIS_313-11521/V96 | 29.006 | 100 | CX131/V101 | 28.777 |
