# Supplementary material for: Genomic selection of agronomic traits in hybrid rice using an NCII population
Source: Rice (N Y). 2018 May 10;11:32. doi: 10.1186/s12284-018-0223-4 (PMC5945574; doi:10.1186/s12284-018-0223-4)
Supplement: Supplementary file 4 — Table S5. Estimated variances and predictabilities under additive model and additive-dominance model. (DOCX 14 kb) [file 12284_2018_223_MOESM4_ESM.docx]

Table S5. Estimated variances and predictabilities under additive model and additive-dominance model

|  | Additive Model | | |  | Additive-dominance Model | | | |
| --- | --- | --- | --- | --- | --- | --- | --- | --- |
| Trait | Additive variance | Residual variance | Predictability |  | Additive variance | Dominance variance | Residual variance | Predictability |
| GY | 192.5239 | 73.0344 | 0.4175 |  | 164.5612 | 50.3723 | 70.4045 | 0.4177 |
| TGW | 18.8454 | 0.8678 | 0.8889 |  | 16.9378 | 3.1649 | 0.7147 | 0.8889 |
| PN | 13.0366 | 3.8407 | 0.4275 |  | 13.0366 | 0.0000 | 3.8407 | 0.4276 |
| PH | 1363.7985 | 47.1687 | 0.8712 |  | 1331.3920 | 53.9335 | 43.9543 | 0.8712 |
| SB | 346.2130 | 28.3681 | 0.7319 |  | 315.4328 | 72.1249 | 24.3938 | 0.7319 |
| GN | 5757.1426 | 610.7513 | 0.6614 |  | 5063.5655 | 1451.0362 | 535.3293 | 0.6614 |
| PL | 17.1236 | 1.7846 | 0.7977 |  | 15.8099 | 3.2466 | 1.6097 | 0.7977 |
| PB | 6.9070 | 0.7355 | 0.6860 |  | 6.2505 | 1.5158 | 0.6487 | 0.6860 |
